# Can Hydrolysable Tannins in Diet of Entire Male Pigs Affect Carcass, Pork Quality Traits, Amino and Fatty Acid Profiles, and Boar Taint, Skatole and Androstenone Levels?

**DOI:** 10.3390/ani11030896

**Published:** 2021-03-21

**Authors:** Ivan Bahelka, Ondřej Bučko, Pavol Fľak

**Affiliations:** 1 Faculty of Agrobiology, Food and Natural Resources, Czech University of Life Sciences Prague, 165 00 Prague, Czech Republic; 2 Faculty of Agrobiology and Food Resources, Slovak Agricultural University Nitra, 949 01 Nitra, Slovakia; ondrej.bucko@uniag.sk (O.B.); flak@centrum.sk (P.F.)

**Keywords:** pig, boar taint, tannin, skatole, fatty acids

## Abstract

**Simple Summary:**

The increasing societal concern in animal welfare has changed centuries-old practices in pig breeding. One of the most debatable is the surgical castration of piglets commonly used in many European countries primarily performed to avoid boar taint. Boar taint is an undesirable and unpleasant odor that is released by mature entire male pigs. The two main compounds responsible for boar taint are androstenone and skatole. While androstenone as a steroid is mainly affected genetically, skatole as a product of microbial degradation of the amino acid tryptophan in hind gut can be influenced by nutrition and feeding. Recently, several studies revealed that hydrolysable tannins in the pig diet have the potential to reduce skatole accumulation in fat tissue. Thus, the objective of this study was to determine the effect of tannin administration (1, 2, 3, and 4%—sweet chestnut extract rich in hydrolysable tannins) on skatole as well as androstenone deposition in adipose tissue, and to observe the impact on carcass and meat quality, chemical, amino and fatty acid composition, and boar taint perception. The results showed that tannins in the diet of entire males did not affect chemical composition, androstenone accumulation, amino, and fatty acid profile in subcutaneous tissue. The effect on carcass value was only slight. However, higher doses of tannins (3 and 4%) increased cooking loss, and partially increased (4% dosage) electrical conductivity in *semimembranosus* muscle. Skatole concentration in fat tissue had a tendency to decline after 2–4% administration compared to control group. Similarly, a tendency to drop in boar taint perception using “hot iron” method was found between control and 2%-supplemented pigs. Higher dietary tannins supplementation (3 and/or 4%) increased several saturated (SFA), monounsaturated (MUFA), and polyunsaturated fatty acids (PUFA) in pork.

**Abstract:**

The slaughtering of entire males increases the probability of incidence of tainted pork due to the presence two main compounds—androstenone and skatole. If a surgical castration of young entire male pigs is stopped in the EU countries, fattening of boars is likely to become one of the most commonly used systems in pig farming. Since skatole production and accumulation in fat tissue can be controlled by dietary approaches, several studies have investigated various feed additives to reduce this compound of boar taint. Ones of the most promising is tannins. The aim of this study was to determine the effect of different dietary tannin level supplementation on carcass, pork quality, chemical, amino and fatty acid composition. as well as perception of boar taint and accumulation of skatole and androstenone in adipose tissue. Eighty entire males were randomly distributed to control (T0) and four experimental groups. Control pigs received standard feed mixture (16.8% CP, 13.9 MJ ME) without any tannin supplementation. Experimental pigs received the same diet with administration of 1% (T1), 2% (T2), 3% (T3) and 4% (T4)—sweet chestnut extract rich in hydrolysable tannins for 40 days (from average live weight of 80 kg until slaughter at average weight 122.28 kg ± 5.63 kg). Dietary tannins supplementation did not show any significant effect on chemical composition, cholesterol content, and amino acid composition of muscle as well as fatty acid composition and androstenone accumulation in adipose tissue. A slight or small effect was observed on carcass and meat quality, respectively. Pigs in groups T4 and/or T3-T4 had higher electrical conductivity in *semimembranosus* muscle and cooking loss value compared to T1, T2 or T0, T1, and T2 groups (*p* < 0.05). Tannins in the pig’s diet greatly affected fatty acid profile in meat of entire males. The highest tannin levels (4%) increased concentrations of lauric, myristic, vaccenic, linoleic, total PUFA, and n-6 PUFA in muscle compared to the control. Similar results were found in group T3 except for vaccenic, linoleic, and total PUFA. On the contrary, concentrations of heptadecanoic and oleic acids in groups T3 and T4 were lower than those in T1 and T2 groups. Perception of boar taint using „hot iron“ method (insertion a hot iron tip of soldering iron into adipose tissue) tended to decrease in T2 group compared with control. Skatole accumulation in fat tissue was reduced in groups T2-T4 at significance level (*p* = 0.052–0.055) compared to the control pigs. In summary, tannins supplementation had no effect on chemical and amino acid composition as well as fatty acid profile in adipose tissue, and only slight on carcass value. However, 4% concentration of tannins significantly increased content of some fatty acids compared to control group.

## 1. Introduction

Avoiding an undesirable odor, so-called boar taint that is released by sexually maturing young boars, as well as improving the pork and sensory quality of their meat are the main reasons for surgical castration of pigs. This technique has been practiced in European pig farming for several centuries. However, increasing concern in animal welfare in the last decade has caused that surgical castration of piglets seems to be unsustainable and is likely to be abandoned in the near future. In 2010, several European countries agreed to voluntarily abandon of surgical castration and are working on suitable alternatives. European Commission strongly supports these activities.

Currently, there are two animal-welfare and economically acceptable alternatives to common surgical practice: Immunocastration and fattening entire males. Immunocastration is based on vaccination against gonadotropin-releasing hormone (GnRH), which leads to inhibition of luteinising hormone (LH) and subsequently elimination of androstenone—one of the two main compounds responsible for boar taint [1,2]. The effect of immunocastration on various aspects of entire male production has recently been the subject of extensive research, e.g., on growth intensity, carcass performance and meat quality [3,4,5,6,7], fatty acid composition in muscle or subcutaneous fat [8,9,10,11], sensory characteristics and consumer´s acceptance of meat [12,13], or boar taint compounds [14,15]. Fattening entire males might also become a suitable alternative because of some advantages of boars over surgical castrates such as greater growth intensity, better feed conversion, and higher lean meat content in carcass. However, slaughtering the boars increases the risk of higher incidence of boar taint, and thus dissatisfaction of consumers with such meat. Therefore, suitable methods for boar taint detection and reduction of its levels in pork need to be developed.

Boar taint is primarily caused by two compounds: Androstenone [16] and skatole [17]. Androstenone is a steroid synthesized in the Leydig cells in the testicles of uncastrated boars. It acts as pheromone in male mammals. In boars, androstenone is transported to the salivary gland. During sexual excitement, it is excreted into saliva and causes so-called immobility reflex (heating) of sow in estrus. Androstenone has an odor like urine or sweat and is perceived by about two-thirds of the human population. It is mainly affected by genetic background and puberty stage and to a lesser extent by environmental factors such as nutrition and feeding, housing conditions, management, etc. Therefore, it is generally accepted that dietary manipulations have a less importance in the production and accumulation of androstenone. Nevertheless, a few studies also reported positive (though insignificant) effect, of dietary supplementation of feed additives on androstenone levels [18,19,20,21,22].

Skatole is a product of microbial degradation of amino acid tryptophan in the large intestine of pigs. It is absorbed from the intestine into blood and through portal vein transported to the liver where is metabolized to a variety of metabolites [23]. In case of high concentration of skatole in blood stream, it is transported and accumulated in the fatty tissue [24]. Unlike androstenone, skatole levels are affected mainly by environmental factors. Dietary composition, e.g., amount of tryptophan or energy sources for microbial activity, may influence intestinal skatole synthesis [25] due to changing the rate of apoptosis, thus reducing the availability of tryptophan in the colon. Nutrition also can change composition of microbiota in large intestine [26], passage of feed through the digestive system, and absorption and metabolism of skatole in the liver.

Recently, several studies have been conducted to reduce skatole levels by nutritional manipulations. Promising results have been achieved by supplementation of boar´s diet with a variety of feed additives such as chicory root or inulin [27,28,29,30,31,32,33,34,35,36], raw potato starch [24,37,38,39], sugar beet pulp [40], Jerusalem artichoke [41], oligofructose and fructooligosaccharides [31,42], and tannins [18,19,43]. The last one is the group of natural astringent polyphenolic compounds widely distributed in many species of plants, where they play a role in protection from predation and help in regulating plant growth [44,45].

Generally, tannins can be divided into (i) condensed and (ii) hydrolysable. Although they were primarily considered to have an anti-nutritional effect on digestibility of nutrients from feed, and then reduction of growth intensity of pigs [46,47], recent research has showed that both condensed and hydrolysable may have harmful or beneficial effects depending on their structure and concentrations [48]. Extract from sweet chestnut wood (*Castanea sativa* Mill.), consisting mainly of hydrolysable tannins, is commonly used in animal nutrition, especially in piglets, to prevent diarrhea after weaning [49,50,51,52,53,54,55].

Recent studies found that hydrolysable tannins can inhibit the activity of bacteria in colon [56], cells proliferation, and apoptosis. Reduced microbial activity resulting from a lesser disponibility of tryptophan and cell debris in the hindgut may lead to lowering the intestinal skatole production [57]. These findings are of considerable interest, especially in the fattening of uncastrated male pigs that might become a suitable alternative to current pork production from castrated males.

The aim of this study was to evaluate the effect of supplementation of hydrolysable tannins on carcass value, chemical composition, and pork quality as well as amino acid and fatty acid profile in uncastrated male pigs. Compared to another work, the present study used shorter time and higher dose of tannins supplementation. Therefore, the effectiveness of this combination in reducing the skatole and/or androstenone has also been the subject of our interest.

## 2. Material and Methods

### 2.1. Animals and Diet

The study was performed in accordance with Act on animal veterinary care No. 39/2007 of Slovak Republic and approved by Animal Care Committee of the Research Institute for Animal Production (RIAP) in Nitra, Slovakia. All pigs (*n* = 80)—progeny of Landrace sows and Yorkshire x Pietrain boars—were housed under conditions of the experimental farm of RIAP. At the average live weight of 65 ± 3.27 kg and age of 123 ± 4.42 days (average ± standard deviation), entire males were housed by pairs/pen and after 2 weeks of transitional period (when fed a control diet) were randomly allocated within litters to the five groups with different levels of tannin supplementation. Control group (T0, *n* = 16) received feed without supplementation; experimental groups (each of 16) were fed the same diet supplemented with 1% (T1), 2% (T2), 3% (T3) and 4% (T4) of Farmatan (Product Feed a.s., Luzianky, Slovakia—official distributor of Tanin Sevnica d.d., Sevnica, Slovenia)—sweet chestnut extract rich in hydrolysable tannins (Table 1). The content of tannins in this product was 75%.

After adaptation period (2 weeks), pigs reached average live weight of 80 kg and started dietary supplementation for 40 days until slaughter at the average weight of 122.28 kg ± 5.63 kg. All animals had ad libitum access to feed using automatic feeding system (Schauer s.r.o., Nitra, Slovakia) and drinking water.

### 2.2. Slaughtering, Carcass and Meat Quality Measurements

Entire males were slaughtered at average live weight of 122.28 kg ± 5.63 kg in one batch at the experimental slaughterhouse of RIAP Nitra by method of electrical stunning (90–100 V, 0.9–1.0 A, 50 Hz) followed by exsanguination. Evisceration was completed about 20 min *post mortem*. Carcasses were classified by ZP (Zwei-punkte) method for lean meat content, backfat thickness at three points (over the 1st, last *thoracic*, and 1st *sacrum vertebra*) was taken and pH (pH_1_), as well as electrical conductivity (EC_1_) were measured 45 min *post mortem* in *longissimus dorsi* (LD) between 13rd–14th rib and *semimembranosus* (SM) muscles using METTLER TOLEDO (pH meter FiveGo™, Columbus, USA) device with combined electrode and BIOTECH (USA), respectively. Chilling of the carcasses (air temperature 2–4 °C, velocity 0.5–1.0 m.s^−1^) started approximately 60 min after slaughter and was continued overnight. After 24-h chilling of the carcasses at 4 °C, measurements of pH (pH_24_) and electrical conductivity (EC_24_) at the same points as reported for *longissimus dorsi* and *semimembranosus* muscle were performed. Carcass weight (CW) and weight of right-half carcass were determined and subsequently, the detailed dissection of right-half carcass into the valuable primal cuts was performed. Valuable meat cuts (VMC) were calculated as summary of neck, shoulder, loin and ham weight; proportion of VMC (PVMC) as weight of VMC from half carcass weight; proportion of ham as weight of ham from CW; proportion of fatty cuts (PFC) as weight of fatty cuts (fat from loin, neck, shoulder, ham, leaf fat) from CW; and proportion of less valuable cuts (PLC) as weight of less valuable cuts (front and hind feet, front and hind totters, head, sacrum bone). Simultaneously, LD muscle (150 g of sample) was removed from the right side of carcass and sliced into 2.5 cm thick chops for further meat quality and chemical composition analysis. One wrapped sample was stored for 4 days at 4 °C for shear force measurements. Colour (L*—lightness, a*—redness, b*—yellowness) was measured by spectrophotometer MINISCAN XE Plus (Hunter Associates Laboratory, Inc., Reston, VA, USA). Water holding capacity (WHC) was analyzed using method of Grau-Hamm modified by Hašek and Palanská [58]. The cooking loss value was determined when the core temperature of the sample reached 80 °C. After that, steak was moved from grill, weighed, and the weight was compared with weight of raw steak before grilling. Drip loss was assessed 24 h after slaughter by Honikel method [59]. Four days after slaughter, the shear force was measured using TEXTURE ANALYSER TA-XT2i device (Stable Micro Systems Ltd., Surrey, UK) reported as tenderness on cooked samples (core temperature 80 °C, time 20 min).

### 2.3. Chemical Composition, Amino and Fatty Acids Profile

Samples from the neck (at level of 5th *thoracic vertebra*) and from adipose tissue (over the neck, each of approximately 200 g) were taken 24 h postmortem and transported to the Chemical laboratory of the Slovak Agricultural University for basic chemical composition, cholesterol content as well as amino acid and fatty acid composition. Each sample was homogenized and subsequently analyzed by the Fourier Transform Infrared (FTIR) method [60] using the device Nicolet 6700 (IET Ltd., Mundelein, IL, USA). Fatty acids were analyzed as percentage of FAME—fatty acid methyl ester.

### 2.4. Feed Analyses

The samples of feed mixture were taken twice during experiment, at the beginning and at the end. After pooling and milling on a 1 mm sieve, the content of dry mater was determined by drying the samples at 105 °C for 3 h. The N content was analyzed using Kjeldahl procedure (Leco FP-2000, Leco, Mönchengladbach, Germany), and crude protein was calculated as N x 6.25 (STN ISO 20483). Crude fiber was determined after digestion with H_2_SO_4_ and KOH, washed with acetone, dried at 130 °C, and then ashed (ČSN ISO 5498). Crude ash content was analysed by combastion at 550 °C (ČSN 467092). Crude fat was determined by acidic hydrolysis as petrol ether extract (ČSN ISO 11085).

### 2.5. Boar Taint, Skatole and Androstenone Determination

Boar taint level was determined 45 min after slaughter on the half carcass. Backfat on level of the last *thoracic vertebra* was heated by inserting the hot iron tip (soldering iron) and smell was evaluated by three previously trained persons sensitive to the odor of skatole and androstenone (modificated Aluwé et al., 2012 [61]). The level of boar taint was evaluated as 0—no, 1—mild or 2—strong. After that, sample of backfat (50 g) was removed from carcass half, moved to the sensory laboratory, inserted into water, heated, and level of boar taint was evaluated at boiling point by the same way. The samples of adipose tissue (100 g) from a part of the belly were taken on the second day after slaughter and stored at −20 °C until analyses. Analysis of androstenone and skatole concentrations in fat tissue was performed in the laboratory of EKOLAB s.r.o. (Košice, Slovakia). The limit for detection was value of 0.02 µg/g for androstenone and 0.01 µg/g for skatole.

### 2.6. Statistical Analysis

The statistical analysis was performed by the statistical package Statistix 9 [62]. Differences among groups (T0–T4) were tested using one-way analyses of variance with fixed effects. Comparison was performed with the Bonferroni multiple comparison method [63]. The values are expressed as mean with standard error of the mean (SEM).

## 3. Results

### 3.1. Carcass Value and Meat Quality

No major influence of tannin supplementation on carcass traits with the exception of percentage of ham was observed (Table 2). Entire males in group T3 had significantly lower (*p* < 0.05) values compared to control group T0.

The effect of dietary tannins on meat quality traits was limited to the electrical conductivity measured 45 min (EC_1_) and/or 24 h (EC_24_) postmortem in *semimebranosus* muscle, and cooking loss (Table 3). Group T4 had higher EC_1_ compared to T1 and T2 groups by 0.77 and 0.73 µS, respectively. Greater differences were found in *musculus semimembranosus* measured 24 h postmortem. Entire males in control T0 and supplemented T4 groups expressed higher values compared to group T1 by 1.69 and 1.54 µS (*p* < 0.01), and to group T2 by 1.43 and 1.28 µS (*p* < 0.05). Interestingly, tannin supplementation with 3 and 4% affected negatively cooking loss. Pigs in these two groups (T3, T4) had significantly greater loss (*p* < 0.01) than T0, T1, and T2 groups in the range 31–47% and 34–50%, respectively.

### 3.2. Chemical Composition, Amino and Fatty Acid Profiles

**Figure 1:**
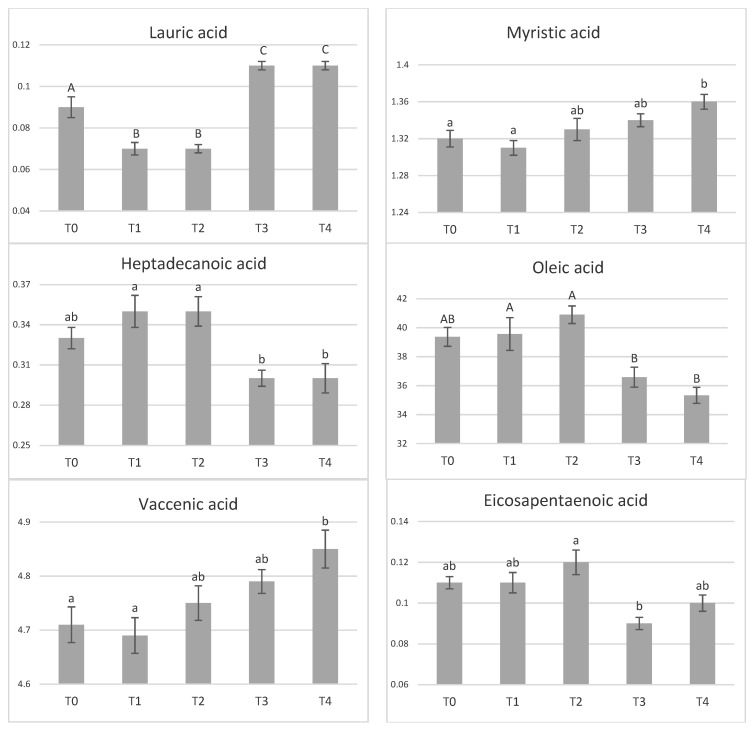

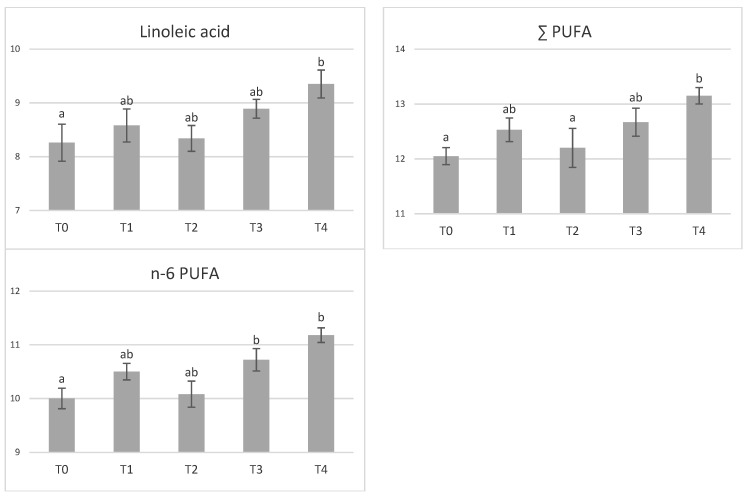
The effect of tannin supplementation on fatty acid profile of muscle. Fatty acids only significantly influenced (*p* < 0.05) are shown. T0—control group, T1—1% tannin supplementation, T2—2% tannin supplementation, T3—3% tannin supplementation, T4—4% supplementation, ^a,b^—values with different letters within rows are significantly different at *p* < 0.05; ^A,B^—values with different letters within rows are significantly different at *p* < 0.01.

Dietary tannin supplementation had no effect on chemical composition of meat (Table 4). Similarly, amino acid profile of *longissimus dorsi* muscle was not affected by tannin supplementation (Table 5). In the adipose tissue, the effect of tannin supplementation on fatty acid profile was not observed (*p* > 0.05), (Table 6). However, significant differences between groups were found in the content of some fatty acids in muscle (Figure 1). The highest tannin dose (T4) increased concentrations of myristic acid compared with control (*p* < 0.05) and T1 (*p* < 0.01) groups. Similarly, both higher tannin supplementations (T3–T4) increased (*p* < 0.01) content of lauric acid compared to the control while lower doses (T1–T2) decreased its content (*p* < 0.01). Concentration of heptadecanoic acids was greater in T1- and T2-supplemented pigs compared with T3 and T4 (*p* < 0.05). At the same time, level of oleic acid was increased in T0-, T1-, and T2-supplemented pigs by 7–14% compared to T3 and T4 groups. The concentration of vaccenic acid was the highest in T4-supplemented pigs and significantly different than control (*p* < 0.05) and T1 pigs (*p* < 0.01). The highest differences between pigs groups were detected in polyunsaturated fatty acids. The highest tannin supplemented entire males (T4) had 1.13, 1.09 and 1.12 times greater proportions of linoleic, total polyunsaturated fatty acids (PUFA), and n-6 PUFA in comparison to unsupplemented pigs (*p* < 0.05). In case of n-6 PUFA, significant difference was detected also between T3 and T0 (*p* < 0.05).

### 3.3. Boar Taint, Androstenon and Skatole Levels

**Figure 2:**
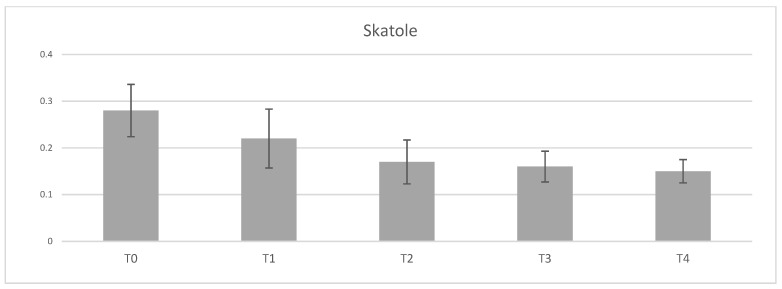
Effect of tannin supplementation on skatole accumulation in adipose tissue. T0—control group, T1—1% tannin supplementation, T2—2% tannin supplementation, T3—3% tannin supplementation, T4—4% supplementation.

The supplementation with tannins did not show any significant impact on androstenone concentration in subcutaneous fat of entire males (Table 7). On the contrary, tannin supplementation had a positive effect on reduction of skatole concentration in fatty tissue. This progressive reduction was observed with increasing supplementation of tannins in pig´s diet. A tendency to significant differences (*p* = 0.052) in supplemented groups T2, T3, and T4 compared to control group (Figure 2) was found. Evaluation of boar taint perception using “hot iron” method also showed a tendency (*p* = 0.054) to significant reduction in group T2 compared to control pigs (Table 7). In contrast, evaluation of boar taint by “boiling” method was not affected with tannin supplementation.

## 4. Discussion

Traditional pork production system based on diet rich in tannins such as acorns and chestnut are used in some Mediterranean countries [64,65]. These fruits containing more than 4% of hydrolysable tannins serve as the important energy source for fattening pigs of specialized native breeds in extensive pig farming. That was the reason for deciding to use the dose of up to 4% of tannins per kg of feed in the present study. As known, tannins ability to bind proteins can be considered a harmful effect in monogastric animals including pigs. This property to bind protein is associated with the antinutritional effects of tannins presenting in decreasing feed palatability and nitrogen digestibility [66,67,68]. Tannins in diet are capable of inducing elevated proline rich proteins (PRP) content in the saliva. Binding capacity of this PRP is directly related to its proline content. Pigs, wild and domestic, have developed this defense strategy to prevent intoxication with diets rich in hydrolysable tannins [69,70]. On the contrary, tannins also show positive properties such as antioxidant, antibacterial, and antidiarrheal, especially important in piglets after weaning [49,50,51,52,53,54].

Carcass value of slaughtered entire males in the present study was not affected by tannin supplementation except for one parameter—percentage of ham. This trait was lower in T3 group compared to control. As other carcass traits did not show any significant differences among tannin-supplemented and control pigs, it can be summarized that dietary tannin administration did not have an adverse impact on carcass quality. No significant effect of tannins on carcass value have been reported in other studies using similar concentrations of tannins (1.5, 3%) in diets and longer period of supplementation (60–98 days) [19,71,72].

In the present study, tannin-supplemented diet did not affect the chemical composition of pork. This finding is in agreement with previous research [73]. Meat quality in our study was partially influenced by tannin supplementation. However, results are quite inconsistent. It seems that increasing the number of tannins in diet resulted in higher electrical conductivity in *semimebranosus* but not in *longissimus dorsi* muscle. Differences between muscles can be explained by different muscle tissue composition, and then resulting different metabolism. Higher cooking loss were also found after 3 and 4% supplementation compared to lower diet doses as well as control group. It seems that higher cooking loss in 3- and 4%-supplemented groups may be related to greater conductivity. However, hypothesis of increasing the electrical conductivity with enhancing tannin supplementation is questioned since a higher value was also found in control group compared to 1- and 2%-supplemented. Therefore, further research is needed to elucidate the mechanism of the effect of tannin supplementation on meat quality traits. The lack of effect of hydrolysable tannins on meat quality has been reported in the other studies with tannins [56,73] as well as studies with other dietary approaches, e.g., inulin or raw potato starch [27,74]. On the contrary, Tretola et al. [43] observed lower meat color (L^*^) and WHC as well as greater cooking loss in pigs supplemented with tannins compared to unsupplemented.

To the best of our knowledge, the amino acid composition of meat from entire males supplemented with tannins has not yet been studied. Similar to basic chemical composition in our study, dietary treatment with tannins did not result in any important differences in observed amino acids between the pig groups.

Related to the fatty acid profile, while no major differences were found in subcutaneous fat, pronounced differences in intramuscular fat were observed. Dietary tannins influenced lauric (C12:0), and myristic (C14:0) acid that were present in a higher proportion in T3 and T4 supplemented groups compared to T0, T1 =, or T2. A similar trend was observed in monounsaturated fatty acids (MUFA) vaccenic acid (C18:1 11c 15t) that was higher in T4 compared to T0 and T1. On the other hand, lower tannin concentrations (T1, T2) resulted in higher accumulation of heptadecanoic (C17:0) and olei acid (C18:1) than those of T3 and T4 supplementation. The most important differences in fatty acid composition were detected in polyunsaturated acids where higher tannin doses, especially T4, resulted in increased linoleic, n-6, and total PUFA amounts compared to the control group. It is debatable if higher accumulation of some SFA and MUFA at T3 and T4 levels were due to direct or indirect impact of tannins in the diet as increasing backfat thickness (even though inconclusive) in the carcass of both two higher-supplemented groups was observed. Interestingly, simultaneous increase in both SFA and PUFA at higher tannin supplementations is unexpected. It is obvious that increase in PUFA (as well as n-6) can be attributed to higher accumulation of linoleic acid. So, it is possible that tannins may affect in some way the metabolism of single fatty acids in pig body. In contrast to our findings, Rezar et al. [73] reported great differences in subcutaneous fat and almost none in intramuscular fat. Tretola et al. [43] did not find any differences in fatty acid composition either in subcutaneous or intramuscular fat after tannin supplementation.

In general, skatole as one of the two most important compounds responsible for boar taint is mainly affected by environmental factors such as housing conditions (type of floor, temperature, air velocity, and humidity), nutrition, feeding, etc. The concentration of skatole in adipose tissue is a result of variety of processes such as formation, absorption, metabolism, and deposition. The crucial role in accumulation of skatole in fat is associated with the efficiency of hepatic metabolism. The potential impact of tannin supplementation on skatole metabolism has not yet been studied. Lower skatole concentrations are associated with higher activities of digestive enzymes of CYP450 family such as 2E1, 2A19, 1A1, 1A2, etc. [18,75]. Despite that, results of various studies are controversial as some report reduction of skatole deposition after tannin-rich diet feeding at higher doses (2 and 3% of tannins) [18,19] while others did not find any positive effect of tannins [43,56]. The common element of all the above-mentioned studies was the supplementation of 1–3% tannins for a longer time period (60–77 days) compared to the present study where the period of tannins administration was only 40 days. As results showed, there was a tendency for reduced skatole accumulation in adipose tissue in groups supplemented with 2, 3, and 4% tannins compared to the control group. That was partially confirmed by the evaluation of boar taint using „hot iron“ method when group T2 also had tendency to lower perception compared to unsupplemented pigs. Regarding effect of tannins on androstenone, supplementation of these additives did not find any impact, even though the two highest doses had higher (although inconclusive) androstenone concentrations in fat tissue than control group. It is in agreement with other studies [43,56].

## 5. Conclusions

Based on the results of this study, dietary tannin supplementation had no effect on chemical and amino acid composition of the muscle, fatty acid profile of subcutaneous tissue and only slight effect on carcass value. However, tannins can affect some pork quality traits. Higher concentrations of tannins (3–4%) increased cooking loss and partially (4%) electrical conductivity of pork. Further, tannins greatly influenced fatty acid composition in muscle. Higher supplementation with 3 and/or 4% of tannins increased several SFA, MUFA, and PUFA in pork. This effect could be beneficial related to increased linoleic, total PUFA and n-6 PUFA but not increased lauric, myristic as well as decreased oleic content. Supplementation with tannins had no effect on androstenone accumulation in fat tissue while there was a tendency to reduction of skatole at higher doses (2–4%). Summarizing, an increase in some polyunsaturated fatty acids and tendency to lower skatole accumulation in adipose tissue can be considered a positive effect of tannin supplementation. In contrast, deterioration of some pork quality traits as well as increasing some saturated fatty acids at higher tannin doses are undesirable from both the meat industry and consumers point of view.

## Figures and Tables

**Table 1:** Ingredients and chemical composition of diets.

Items	T0	T1	T2	T3	T4
Ingredients, g/kg					
Wheat	150	150	150	150	150
Barley	360	360	360	360	360
Corn	150	150	150	150	150
Wheat bran	80	70	60	50	40
Soybean meal	110	110	110	110	110
Rapeseed meal	100	100	100	100	100
Tannin	0	10	20	30	40
Mineral suppl.	25	25	25	25	25
Premix	10	10	10	10	10
Ground limestone	10	10	10	10	10
Fodder salt	5	5	5	5	5
Analysis, g/kg					
DM	898.4	896.3	898.8	899.0	895.8
Crude protein	168.4	167.6	167.8	164.7	165.2
Crude fibre	50.8	51.6	49.9	51.4	50.5
Crude fat	27.2	26.5	25.5	26.4	26.7
Crude ash	43.7	45.8	44.9	43.3	44.7
ME, MJ/kg	13.9	13.8	13.8	13.7	13.8

DM—dry matter, ME—Metabolisable energy. T0—control group, T1—1% tannin supplementation, T2—2% tannin supplementation, T3—3% tannin supplementation, T4—4% supplementation.

**Table 2:** Carcass value of control (T0) and tannin-supplemented (T1–T4) groups.

Trait	Treatment					SEM	*p*-Value
	T0	T1	T2	T3	T4		
Slaughter weight, kg	121.19	122.80	122.10	121.59	121.63	0.319	0.642
Carcass weight, kg	94.56	95.21	95.55	94.42	94.74	0.461	0.524
Half carcass, kg	45.20	45.65	46.59	45.45	45.34	0.623	0.213
BT, mm	21.94	22.80	25.73	25.10	25.06	0.425	0.322
VMC, kg	24.11	23.96	24.43	23.54	23.74	0.186	0.637
PVMC, %	53.38	52.48	52.45	51.74	52.37	0.569	0.593
ZP-method, %	58.65	58.94	57.82	57.77	57.51	0.255	0.438
Percentage of ham, %	20.99 ^a^	20.65 ^ab^	20.47 ^ab^	19.76 ^b^	20.37 ^ab^	0.216	0.043
PFC, %	12.35	12.37	12.80	13.37	13.21	0.286	0.568
PLC, %	15.68	15.64	15.16	15.67	15.56	0.824	0.622

T0—control group, T1—1% tannin supplementation, T2—2% tannin supplementation, T3—3% tannin supplementation, T4—4% supplementation, BT—backfat thickness as average of three measurements, VMC—valuable meat cuts, PVMC—percentage of valuable meat cuts, PFC—percentage of fatty cuts, PLC—percentage of less valuable cuts. ^a,b^—values with different letters within rows are significantly different at *p* < 0.05.

**Table 3:** Meat quality of control (T0) and tannin-supplemented (T1–T4) groups.

Trait	Treatment					SEM	*p*-Value
	T0	T1	T2	T3	T4		
pH_1_ LD	6.37	6.54	6.41	6.20	6.28	0.039	0.097
pH_1_ SM	6.25	6.46	6.36	6.24	6.28	0.031	0.188
pH_24_ LD	5.99	6.07	6.07	6.01	6.03	0.025	0.816
pH_24_ SM	6.01	6.02	6.10	6.05	6.07	0.021	0.710
EC_1_ LD, µS	1.83	1.64	1.74	2.07	2.34	0.083	0.056
EC_1_ SM, µS	2.01 ^ab^	1.64 ^a^	1.68 ^a^	2.11 ^ab^	2.41 ^b^	0.080	0.015
EC_24_ LD, µS	2.01	1.45	1.67	2.07	2.24	0.115	0.246
EC_24_ SM, µS	3.98 ^Aa^	2.29 ^B^	2.55^b^	3.43 ^ab^	3.83 ^Aa^	0.143	<0.001
Colour L*	50.58	50.45	50.60	51.55	52.21	0.491	0.741
a*	1.39	0.60	1.40	1.25	1.05	0.090	0.062
b*	8.06	7.82	8.16	8.33	7.97	0.128	0.785
WHC, %	36.38	35.40	33.70	37.12	32.33	0.690	0.118
Shear force, g	11 142	10 889	10 777	12 184	11 794	346.7	0.667
Cooking loss, %	24.01 ^A^	21.40 ^A^	23.42 ^A^	31.44 ^B^	32.09 ^B^	0.862	<0.001
Drip loss, %	3.74	3.20	2.95	4.29	3.97	0.176	0.130

T0—control group, T1—1% tannin supplementation, T2—2% tannin supplementation, T3—3% tannin supplementation, T4—4% supplementation, LD—*longissimus dorsi* muscle, SM—*semimembranosus* muscle, EC—electrical conductivity, Colour L*—lightness, a*—yellowness, b*—redness, WHC—water holding capacity. ^a,b^—values with different letters within rows are significantly different at *p* < 0.05. ^A,B^—values with different letters within rows are significantly different at *p* < 0.01.

**Table 4:** Basic chemical composition and cholesterol content of *musculus longissimus dorsi* in control (T0) and tannin-supplemented (T1–T4) groups.

Trait	Treatment					SEM	*p*-Value
	T0	T1	T2	T3	T4		
Total water, %	72.45	72.14	72.33	71.95	72.18	0.111	0.624
Crude protein, %	25.19	25.27	25.36	25.54	25.81	0.083	0.095
Crude fat, %	0.94	0.92	0.80	0.87	0.65	0.037	0.077
Cholesterol, %	0.35	0.35	0.33	0.37	0.33	0.007	0.297

T0—control group, T1—1% tannin supplementation, T2—2% tannin supplementation, T3—3% tannin supplementation, T4—4% supplementation.

**Table 5:** Amino acids composition of LD muscle in control (T0) and tannin-supplemented (T1–T4) groups.

Trait	Treatment					SEM	*p*-Value
	T0	T1	T2	T3	T4		
Arginine, %	1.26	1.29	1.27	1.18	1.22	0.020	0.394
Cysteine, %	0.30	0.30	0.30	0.28	0.28	0.004	0.136
Phenylalanine, %	0.81	0.83	0.82	0.75	0.77	0.012	0.201
Histidine, %	0.87	0.87	0.85	0.77	0.78	0.017	0.129
Isoleucine, %	0.77	0.78	0.77	0.72	0.74	0.013	0.447
Leucine, %	1.58	1.61	1.59	1.46	1.51	0.024	0.279
Lysine, %	1.68	1.72	1.69	1.57	1.62	0.027	0.365
Methionine, %	0.65	0.65	0.65	0.61	0.62	0.010	0.303
Threonine, %	0.91	0.92	0.91	0.84	0.87	0.013	0.183
Valine, %	0.86	0.88	0.87	0.81	0.82	0.010	0.061

T0—control group, T1—1% tannin supplementation, T2—2% tannin supplementation, T3—3% tannin supplementation, T4—4% supplementation.

**Table 6:** Fatty acids ^1^ composition of adipose tissue in control (T0) and tannin-supplemented (T1–T4) groups.

Trait	Treatment					SEM	*p*-Value
	T0	T1	T2	T3	T4		
Myristic C14:0	1.24	1.23	1.24	1.29	1.24	0.008	0.115
Palmitic C16:0	24.16	24.19	24.52	24.86	23.97	0.131	0.160
Stearic C18:0	11.85	11.80	12.40	12.50	11.67	0.194	0.536
∑SFA	39.59	39.67	40.46	40.83	39.39	0.299	0.440
Oleic C18:1 n-9	40.58	40.90	40.28	39.58	40.31	0.193	0.266
∑MUFA	48.19	48.53	47.76	47.03	47.92	0.237	0.328
Linoleic C18:2 n-6	9.43	9.39	9.31	9.23	9.74	0.091	0.405
Linolenic C18:3 n-3	0.63	0.62	0.62	0.62	0.67	0.008	0.127
∑PUFA	10.89	10.80	10.75	10.60	11.19	0.114	0.504
n6 FA	9.67	9.60	9.61	9.47	10.01	0.099	0.436
n3 FA	0.72	0.72	0.72	0.70	0.75	0.008	0.327

^1^ Fatty acids are analysed as % of FAME—fatty acid methyl ester, ns—no significance, T0—control group, T1—1% tannin supplementation, T2—2% tannin supplementation, T3—3% tannin supplementation, T4—4% supplementation.

**Table 7:** Effect of tannins on androstenone and skatole accumulation, and perception of boar taint.

Trait	Treatment					SEM	*p*-Value
	T0	T1	T2	T3	T4		
Androstenone, µg/g	0.79	0.43	0.62	0.80	0.84	0.064	0.321
Boar taint—“hot iron”	1.94 ^‡^	1.40	1.10 ^‡^	1.53	1.56	0.091	0.054
Boar taint—“boiling”	1.81	1.40	1.40	1.71	1.69	0.090	0.550

T0—control group, T1—1% tannin supplementation, T2—2% tannin supplementation, T3—3% tannin supplementation, T4—4% supplementation. ^‡^ *p* = 0.054
